# Some effects of domain size and boundary conditions on the accuracy of airfoil simulations

**DOI:** 10.1186/s42774-023-00163-z

**Published:** 2024-03-01

**Authors:** Narges Golmirzaee, David H. Wood

**Affiliations:** Department of Mechanical and Manufacturing Engineering, University of Calgary, Calgary, T2L 1Y6 Alberta Canada

**Keywords:** Airfoil simulations, Boundary conditions, Incompressible flow, Aerodynamic forces, Vorticity, Impulse equation

## Abstract

This paper investigates a specific case of one of the most popular fluid dynamic simulations, the incompressible flow around an airfoil (NACA 0012 here) at a high Reynolds number ($6 \times 10^6$). OpenFOAM software was used to study the effect of domain size and four common choices of boundary conditions on airfoil lift, drag, surface friction, and pressure. We also examine the relation between boundary conditions and the velocity, pressure, and vorticity distributions throughout the domain. In addition to the common boundary conditions, we implement the “point vortex” boundary condition that was introduced many years ago but is now rarely used. We also applied the point vortex condition for the outlet pressure instead of using the traditional Neumann condition. With the airfoil generating significant lift at incidence angles of $5^\circ , 10^\circ$, and $14^\circ$, we confirm a previous finding that the boundary conditions combine with domain size to produce an induced (pressure) drag. The change in the pressure drag with domain size is significant for the commonly-used boundary conditions but is much smaller for the point vortex alternative. The point vortex boundary condition increases the execution time, but this is more than offset by the reduction in domain size needed to achieve a specified accuracy in the lift and drag. This study also estimates the error in total drag and lift due to domain size and shows it can be almost eliminated using the point vortex boundary condition. We also used the impulse form of the momentum equations to study the relation between drag and lift and spurious vorticity, which is generated as a result of using non-exact boundary conditions. These equations reveal that the spurious vorticity throughout the domain is associated with cancelling circulation around the domain boundaries.

## Introduction

Predicting the flow around an airfoil plays an important role in multiple engineering applications, ranging from the design of wind turbine blades, propellers, and wings to turbomachinery and hydrodynamic engineering. Despite being a two-dimensional (2D) problem, airfoil simulation has a number of important challenges.

First, accurate results require using a suitable turbulence model. There are different approaches for solving turbulent flows, e.g., Direct Numerical Simulation (DNS), Reynolds-Averaged Navier-Stokes (RANS), Large Eddy Simulation (LES), and hybrid RANS/LES.

DNS approaches numerically solve the Navier-Stokes equations for motions of all scales without resorting to any turbulence model. They require very fine resolution, making them computationally expensive, particularly when the Reynolds number, *Re*, is high, or the geometry is complex. Nakhchi et al. [1] employed the spectral/*hp* element method version of DNS for an airfoil simulation with *Re* varying from $2.5\times 10^4$ to $1.5\times 10^5$ and the angle of attack, $\alpha$, from $0^{\circ }$ to $16^{\circ }$. They investigated the flow structures and laminar separation bubbles over a National Advisory Committee for Aeronautics (NACA) 4412 wind turbine blade section and noticed that the simulations accurately capture the vortex generation, flow separation points, and pressure fluctuations, which other turbulence models can not effectively capture.

RANS turbulence models divide the flow properties into a mean and a fluctuating part. This approximation makes the simulations faster and more cost-effective, but less accurate at least for high values of $\alpha$ when separation occurs. RANS models are usually less accurate than DNS. The most well-known RANS turbulence models are the Spalart-Allmaras (SA), $k-\epsilon$ and $k-\omega$ SST. Martini et al. [2] simulated a fluid flow at $Re=8.64\times 10^6$ around both a clean and an iced NACA 64-618 airfoil using SA and $k-\omega$ SST models. They showed that the two models give similar results for the lift and drag, which agreed with experiments for the clean airfoil for low values of $\alpha$. The $k-\omega$ SST model was more accurate in the presence of ice and strong reverse pressure gradients. On the other hand, they reported that the SA model has drawbacks in case of high values of $\alpha$.

LES directly resolves large eddies, which are more complex, and models the fluctuating motion of smaller scales. It is more cost-effective than DNS and more accurate than RANS. Ziadé et al. [3] studied a flow with a low *Re* of $1\times 10^5$ around a NACA 0025 airfoil at $\alpha =5^{\circ }$ and $12^{\circ }$ using LES and compared the simulation results with experimental data. Based on their work, LES can accurately predict the locations of flow separation, transition, and reattachment.

RANS/LES approach is more useful when flow conditions are complex. It switches between the RANS and LES models and optimizes the accuracy and computational efficiency. Tangermann and Klein [4] employed two hybrid RANS/LES models, DDES and IDDES, to study laminar separation on a NACA 0018 airfoil at $\alpha =4^{\circ }$ and $Re=80000$. Their work highlighted the significance of mesh resolution and discretization schemes as two influencing factors on flow separation and transition. They also reported that for flows with laminar separation, using IDDES is more accurate and preferable.

This work is restricted to simulating steady flows using RANS models to investigate the effect of the boundary condition (BC) and domain size on the accuracy of results with the expectation that the outcome of this investigation would help improve the boundary conditions for more complex modellings. Since we are not examining the accuracy of different turbulence models, the simulations described here used the SA model only. Besides, our study of cascade flows [5] showed very little differences in the lift and drag between the SA and $k-\omega$ SST turbulence models for the range of $\alpha$ considered here.

Other essential factors to be taken into account are the appropriate domain shape, high-quality mesh, and large enough number of cells. Lu et al. [6] found that a structured mesh gave more accurate drag predictions, but an unstructured mesh gave the lift to a comparable accuracy. Also, they reported that a structured mesh provides better convergence and higher resolution compared to an unstructured mesh. Regarding the domain shape, Lu et al. [6] reported that since the O-mesh cannot accurately capture the wake region, the lift and drag errors are higher for this mesh type compared to the C-mesh and H-mesh. Using a C-mesh with boundaries less than 30*c* from the airfoil, where *c* is the airfoil chord, is a common practice [7–11], but doing a domain independence study is essential to make sure that the boundaries do not affect the results. In this study, we show that using these domain sizes can result in significant errors in the aerodynamic forces.

Another important consideration is to select appropriate BCs. In 2D flows, the velocity disturbances decay asymptotically as the inverse of the distance from the body while they decay as the square of the inverse distance in three-dimensional (3D) flows. Consequently, determining the domain size and the outer or far-field BCs plays a crucial role in achieving accuracy in a 2D simulation when the domain size is comparable to 30*c*. An added complication is that the correct outer BCs for pressure and velocity depend on the solution, and there are no simple physical principles, such as the no-slip condition at the inner or body boundary, that can be used to set them for airfoil flow.

An important velocity disturbance for a lifting airfoil is the circulation given by the Kutta-Joukowski (KJ) theorem. To approximate this disturbance, Thomas and Salas [12] applied the “point vortex” (PV) BC, which comprises simple equations for the velocity perturbations along the domain boundaries due to a vortex placed at the aerodynamic center - the quarter chord position - with a strength given by the KJ theorem. Despite its simplicity, the PVBC is rarely used in modern airfoil simulations. Destarac’s [13] Euler solutions of the flow around a NACA 0012 airfoil showed that reducing the domain size while keeping the same BCs increased the pressure drag. Applying the PVBC reduced the magnitude of the increase, which is proportional to the lift squared (as in 3D) and inversely proportional to the domain size.

The present study extends Destarac’s [13] important insight by showing that the viscous drag varies much less with the domain size. Thus, the error in the pressure drag can be used to estimate the domain size needed to achieve a desired accuracy for the total drag. We demonstrate that the PVBC is easily implemented in a modern CFD software, OpenFOAM, leading to a significantly smaller domain size for a given accuracy than the common BCs. We also consider the associated issue of vorticity generation within the domain and along its boundaries by the choice of BCs. Moreover, an impulse formulation for the lift and drag is used to determine the significance of the spurious vorticity.

In the first part of Section 2, we survey the common BCs used for airfoil simulations and select four for further study. Our implementation of the PVBC is described and the choice of a baseline domain size explained. Section 2 is completed by explaining the choice of computational domain and domain size. Section 3 describes the numerical solutions and a demonstration of grid convergence. Section 4 contains the results and their analysis using the impulse form of the momentum equations. Section 5 lists the conclusions.

## Boundary conditions and domain size

### Boundary conditions

**Fig. 1:**
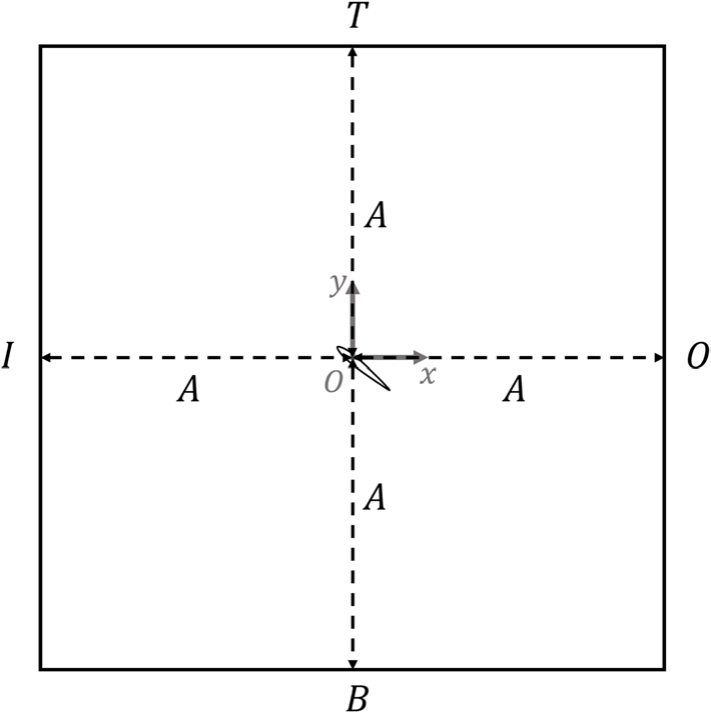
The computational domain, boundary labels, and coordinate system. The flow enters the inlet (I) on the left and exits the outlet (O). The top (T) and bottom (B) boundaries are also identified. The airfoil at the origin is shown for a positive $\alpha$. Note that this domain has 1 unit length in the *z*-direction that is not shown

We employed four BCs that are commonly used for airfoil simulations: BC-1, BC-2, BC-3, and BC-4, as described in Table 1, applied to the velocity vector, $\varvec{U}$, pressure, *p*, turbulent viscosity or eddy viscosity, $\nu _{t}$, and viscosity-like variable, $\tilde{\nu }$ at the inlet (I), top (T), bottom (B), and outlet (O) boundaries of our square domains (Fig. 1). The reason for using rectangular domains rather than C-grids is given at the end of this subsection and Section 2.2. The values for the free-stream velocity, $U_{\infty}$, $\nu _{t}$, and $\tilde{\nu }$ are explained in Section 3.

**Table 1:** Typical boundary conditions used in the airfoil simulations. The CV was the same for all cases

Boundaries	$\varvec{U}\ \mathbf{[ms}^{\varvec{-1}}\varvec{]}$	$\varvec{p}\ \mathbf{[m}^{\varvec{2}}\mathbf{s}^{\varvec{-2}}\varvec{]}$	$\varvec{\nu }_{\varvec{t}}\ \mathbf{[m}^{\varvec{2}} \mathbf{s}^{\varvec{-1}}\varvec{]}$	$\tilde{\varvec{\nu }}\ \mathbf{[m}^{\varvec{2}} \mathbf{s}^{\varvec{-1}}\varvec{]}$
	BC-1			
I, T, B	fixedValue, (51.48, 0, 0)	zeroGradient	fixedValue, 8.58 × 10^−6^	fixedValue, 3.432 × 10^−5^
O	zeroGradient	fixedValue, 0	zeroGradient	zeroGradient
Airfoil	fixedValue, (0, 0, 0)	zeroGradient	fixedValue, 0	fixedValue, 0
	BC-2			
I, T, B, O	freestreamVelocity, (51.48, 0, 0)	freestreamPressure, 0	freestream, 8.58 × 10^−6^	freestream, 3.432 × 10^−5^
	BC-3			
I	fixedValue, (51.48, 0, 0)	zeroGradient	fixedValue, 8.58 × 10^−6^	fixedValue, 3.432 × 10^−5^
T, B	slip	slip	slip	slip
O	zeroGradient	fixedValue, 0	zeroGradient	zeroGradient
	BC-4			
I	fixedValue, (51.48, 0, 0)	zeroGradient	fixedValue, 8.58 × 10^−6^	fixedValue, 3.432 × 10^−5^
T, B	symmetry	symmetry	symmetry	symmetry
O	zeroGradient	fixedValue, 0	zeroGradient	zeroGradient

BC-1 is based on the recommendation of Versteeg and Malalasekera [14]. It comprises the Dirichlet boundary condition for $\varvec{U}$ at I, T, and B and the Neumann boundary condition at O. For the pressure, BC-1 uses the Neumann boundary condition at I, T, and B and a fixed value at O.

One of the OpenFOAM tutorials [15] suggested BC-2 for an airfoil simulation. The “freestream” boundary condition in OpenFOAM is a mixture of the fixed value and the zero gradient boundary conditions. OpenFOAM chooses between these two conditions depending on the sign of the mass flux across the boundary.

BC-3 uses “slip” boundary condition [16] at T and B. The slip boundary condition for vector quantities enforces a zero-gradient condition for the tangential component and a zero value for the normal component. For scalars, it is the same as the “zeroGradient” boundary condition in OpenFOAM.

BC-4, which uses the “symmetry” boundary condition [17] at T and B, can be regarded as analogous to BC-3. It imposes a parallel flow at boundaries as well.

Table 2 lists the three additional BCs we developed, partly through trial and error. The PVBC, which uses the transitional periodic boundary condition [18] at T and B, is easily derived from the Biot-Savart (BS) law. If we decompose the velocity to its unperturbed and induced components, $\vec{U}=(U_{\infty }+u, v)$, then *u* and *v* at a distance *r* from the point vortex are given by

1 $$\begin{aligned} u=\frac{\Gamma }{2\pi r} \sin \theta \qquad \text {and} \qquad v=-\frac{\Gamma }{2\pi r} \cos \theta , \end{aligned}$$

where $\theta$ is the angle between the induced velocity and the *y*-axis, and $\Gamma$ is the circulation, which is defined as

2 $$\mathrm\Gamma=\oint_{C}\vec{U}\cdot\mathrm d\vec l=\oint_{\mathrm\Sigma}\vec{\mathrm\Omega}\cdot\mathrm d\vec{\mathrm\sigma},$$

where $\Sigma$ is the surface whose boundary is *C*, $\mathrm d\vec{\mathrm\sigma}$ is the vector surface element, $\mathrm d\vec l$ is the unit tangent vector to *C*, and $\vec {\Omega }$ is the vorticity vector, $\vec {\Omega }=\nabla \times \vec{U}$.

**Table 2:** The additional boundary conditions used in the present work. The CV was the same as in Table 1

Boundaries	$\varvec{U}\ \mathbf{[ms}^{\varvec{-1}}\varvec{]}$	$\varvec{p}\ \mathbf{[m}^{\varvec{2}}\mathbf{s}^{\varvec{-2}}\varvec{]}$	$\varvec{\nu }_{\varvec{t}}\ \mathbf{[m}^{\varvec{2}} \mathbf{s}^{\varvec{-1}}\varvec{]}$	$\tilde{\varvec{\nu }}\ \mathbf{[m}^{\varvec{2}} \mathbf{s}^{\varvec{-1}}\varvec{]}$
	Point Vortex BC (PVBC)			
I, T, B	Eq. (6)	zeroGradient	fixedValue, 8.58 × 10^−6^	fixedValue, 3.432 × 10^−5^
O	zeroGradient	Eq. (8)	zeroGradient	zeroGradient
	Periodic BC (PeBC)			
I	fixedValue, (51.48, 0, 0)	zeroGradient	fixedValue, 8.58 × 10^−6^	fixedValue, 3.432 × 10^−5^
T, B	cyclicAMI	cyclicAMI	cyclicAMI	cyclicAMI
O	zeroGradient	fixedValue, 0	zeroGradient	zeroGradient
	Point Vortex + Periodic BC (PVPeBC)			
I	Eq. (9)	zeroGradient	fixedValue, 8.58 × 10^−6^	fixedValue, 3.432 × 10^−5^
T, B	cyclicAMI	cyclicAMI	cyclicAMI	cyclicAMI
O	zeroGradient	Eq. (10)	zeroGradient	zeroGradient

The drag and lift coefficients are defined as

3 $$\begin{aligned} C_{\textrm{d}}=\frac{2D}{\rho U^2_{\infty}c} \qquad \text {and} \qquad C_{\textrm{l}}=\frac{2L}{\rho U^2_{\infty } c}, \end{aligned}$$

respectively, where *D* and *L* are the drag and lift per span (the *z*-direction), and $\rho$ is the density of the fluid. Also, one of the most important equations in aerodynamics applications is the KJ theorem, which is

4 $$\begin{aligned} L=\rho U_{\infty } \Gamma \cdot \end{aligned}$$

Substituting the equation for $C_{\textrm{l}}$ into Eq. (4) gives

5 $$\begin{aligned} \Gamma =C_{\textrm{l}}U_{\infty }c/2. \end{aligned}$$

Using Eqs. (1) and (5), the PVBC for the velocity vector is

6 $$\begin{aligned} (U_\mathrm {\infty }+u, v)=\left( U_{\infty }+\frac{ yU_{\infty }C_{\textrm{l}} c}{4\pi (x^2+y^2)}, -\frac{xU_{\infty }C_{\textrm{l}} c}{4\pi (x^2+y^2)}\right), \end{aligned}$$

where $\vec{x}=(x,y)$ is the position vector of an arbitrary point on the boundary measured from the aerodynamic center. Neither Thomas and Salas [12] nor Destarac [13] clearly described their implementation of the PVBC for the pressure. Numerical experiments led us to the following for the outlet. Using the PVBC with Bernoulli’s equation, the pressure is given by

7 $$\begin{aligned} p=-\rho \left( U_{\infty } u+\frac{u^2}{2}+\frac{v^2}{2}\right) \cdot \end{aligned}$$

Ignoring the second order terms gives

8 $$\begin{aligned} p=-\frac{\rho y U_{\infty }^2 C_{\textrm{l}} c}{4 \pi (x^2+y^2)}\cdot \end{aligned}$$

Equation (8) is applied at the outlet. As far as we know, this is the first use of the PVBC for the outlet pressure.

One reason for the unpopularity of the PVBC may be that it is iterative; the value of $C_{\textrm{l}}$ is required in Eqs. (6) and (8). We show below, however, that it is easy to implement the PVBC as defined in Table 2, in OpenFOAM.

Suppose we change perspective and consider the airfoil as one of an infinite cascade of lifting bodies separated vertically by distance 2*A* [5]. In that case, the natural computational domain is rectangular, and the T and B BCs become periodic. This boundary condition is PeBC in Table 2. The lift and drag will be affected by this change [5], but periodic BCs are exact in the sense that the no-slip condition at the airfoil surface is exact, and so PeBC is a useful test case here.

To implement the PV equations for PeBC, we apply periodicity at the T and B boundaries and the following velocity components, which are modified by a straightforward extension of the BS law analysis to sum over all PVs in the cascade

9 $$\begin{aligned} U =&U_{\infty }-\frac{ U_{\infty }C_{\textrm{l}} c \sin (\pi y/A)}{8A [\cos (\pi y/A)-\cosh (\pi x/A)]},\nonumber \\ v =&\frac{U_{\infty }C_{\textrm{l}} c \sinh (\pi x/A)}{8A[\cos (\pi y/A)-\cosh (\pi x/A)]}, \end{aligned}$$

and the pressure at the outlet

10 $$\begin{aligned} p = \frac{\rho U^2_{\infty } C_{\textrm{l}} c \sin ( \pi y/A)}{8 A \left[ \cos ( \pi y/A)-\cosh ( \pi x/A)\right] }\cdot \end{aligned}$$

These equations are used in PVPeBC, the last case listed in Table 2.

The PVBC and PVPeBC were applied using the following procedure. First, we included the “forceCoeffs” function and set the appropriate values for “liftDir”, “dragDir”, “CofR”, “pitchAxis”, “magUInf”, “lRef” and “Aref” in the “controlDict” file. Adding this function generated a file called “coefficient” inside the “postprocessing” folder. The coefficient file stored $C_{\textrm{d}}$ and $C_{\textrm{l}}$ calculated in each time step. At every 100 iterations, $C_{\textrm{l}}$ was used to determine the boundary values of *u*, *v,* and *p* for the next 100 iterations. Changing the determination of $C_{\textrm{l}}$ from every 100 steps to every 1, 200, and 1000 steps was found not to alter the converged results or to have a significant effect on the execution time.

### Computational domain and domain size

A rectangular domain is required for the periodic BCs, so we used this shape for all simulations. A square domain of sides 2*A*, where *A* is a multiple of *c*, has the advantage of specifying domain size by a single parameter, which is useful in estimating the error in $C_{\textrm{d}}$. A rectangular domain also simplifies the application of the momentum equations in Section 4.3.

Many studies of airfoils use domains of dimensions less than 30 far from an airfoil [7–11]. Therefore, $A=30$ was chosen as the base case to determine the appropriate number of cells and begin assessing the various BCs.

For reasons that are explained in Section 4.3, most results were obtained with structured meshes. Results obtained with unstructured meshes will be highlighted as such.

## Numerical method

We used OpenFOAM software [16] for all simulations. The simpleFoam algorithm for incompressible, steady, turbulent flows, was employed together with the Spalart-Allmaras one-equation RANS turbulence model. The aerodynamic center of the airfoil was at the origin of the coordinate system. The simulations were done for a range of angles of attack $\alpha = 5^\circ , 10^\circ$, and $14^\circ$ to examine how variations in the lift influence the accuracy of the boundary conditions. These angles were specifically chosen to avoid flow separation. *c* = 1 m; the freestream velocity, $U_{\infty }$, was 51.48 ms^−1^; the kinematic viscosity, $\nu$, was $8.58 \times 10^{-6}$ m^2^s^−1^. The values for the turbulence viscosity, $\nu _t$, and $\tilde{\nu }$ are within the ranges recommended by Spalart and Rumsey [19], Spalart [20], and Menter [21]. With these values, *Re* was $6 \times 10^6$, which causes turbulent flow over most of the airfoil and so avoids having to simulate transition.

Because it is a common value in modern practice, our initial simulations used $A=30$ combined with BC-1. Without attempting to find the “best” *A*, Golmirzaee and Wood [5] found $A=500$ with BC-1 and the Spalart-Allmaras turbulence model gave results very close to the experimental data from Ladson [22] for the NACA 0012. The computed $C_{\textrm{d}}$ and $C_{\textrm{l}}$ were 0.01220 and 1.07687, respectively, for $\alpha =10^{\circ }$ and their finest grid. Table 3 also presents the experimental data [22] for $C_{\textrm{d}}$ and $C_{\textrm{l}}$. The need to use a large *A* to achieve this agreement was part of the motivation for the present study. We aim to investigate the effect of the domain size and boundary conditions on the force coefficients.

**Table 3:** Experimental data for a NACA 0012 airfoil at different values of $\alpha$, $Re = 5.95 \times 10^6$, Mach number of 0.15 and grit size 80 [22]

	$\varvec{\alpha } \varvec{=} \varvec{4.04}^{\varvec{\circ }}$	$\varvec{\alpha } \varvec{=} \varvec{6.09}^{\varvec{\circ }}$	$\varvec{\alpha } \varvec{=} \varvec{10.12}^{\varvec{\circ }}$	$\varvec{\alpha } \varvec{=} \varvec{14.22}^{\varvec{\circ }}$
$C_{\textrm{d}}$	0.00823	0.00885	0.01201	0.01625
$C_{\textrm{l}}$	0.4316	0.6546	1.0707	1.4365

**Fig. 2:**
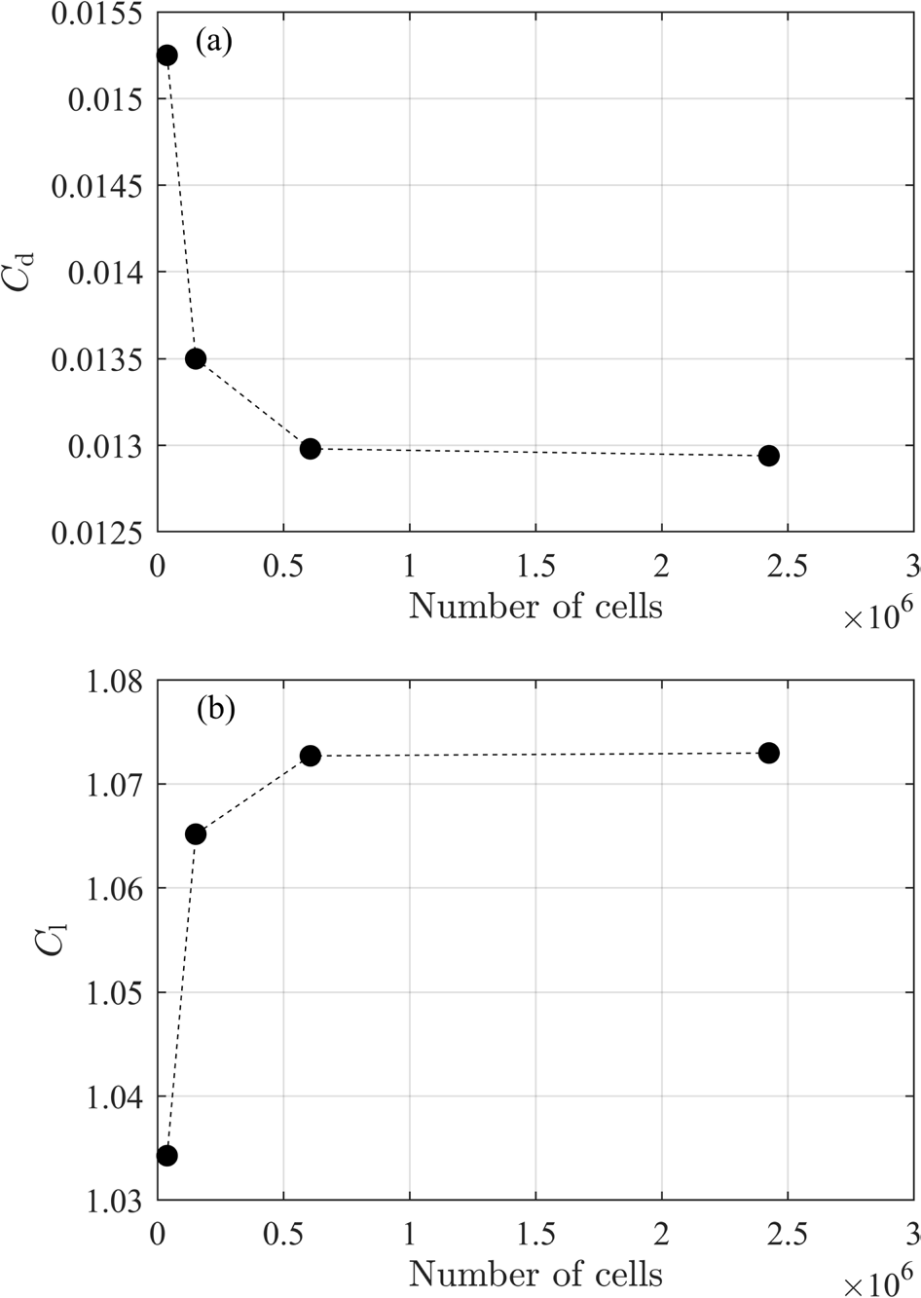
Grid convergence study for a NACA 0012 airfoil at $\alpha =10^{\circ }$, $A=30$, and BC-1; **a** $C_{\textrm{d}}$ vs number of cells, and **b** $C_{\textrm{l}}$ vs number of cells

A grid convergence study is necessary to quantify the numerical uncertainty arising from the choice of the number of cells. Roache [23] proposed a method for quantifying the uncertainty of the grid convergence using Richardson extrapolation. The resulting estimated values for $C_{\textrm{d}}$ and $C_{\textrm{l}}$ at zero grid spacing are 0.012937 and 1.07273 with the numerical uncertainty bounds of $0.03 \%$ and $6 \times 10^{-6} \%$, respectively. Using Roache’s [23] grid convergence index, it was established that the data in Table 4 were in the asymptotic range. Figure 2 also shows the monotonic behavior of $C_{\textrm{d}}$ and $C_{\textrm{l}}$ with the number of cells. We used the fine grid and same turbulence model and only changed the BCs, as explained in Section 2.1.

**Table 4:** $C_{\textrm{d}}$ and $C_{\textrm{l}}$ for a NACA 0012 airfoil at $\alpha =10^{\circ }$ having BC-1, $A=30$ and different numbers of cells, *N*. GR is the growth ratio

Grid	*N*	First grid height	GR	$\varvec{\min }\ \varvec{y}^{\varvec{+}}$	$\varvec{\max }\ \varvec{y}^{\varvec{+}}$	$\varvec{C}_{\textbf{d}}$	$\varvec{C}_{\textbf{l}}$
Coarsest	37,888	$2.16 \times 10^{-6} \ c$	1.75	0.0329	0.9170	0.01525	1.03427
Coarse	151,552	$1.08 \times 10^{-6} \ c$	1.32	0.0175	0.4080	0.01350	1.06517
Intermediate	606,208	$5.4 \times 10^{-7} \ c$	1.15	0.0056	0.2024	0.01298	1.07271
Fine	2,424,832	$2.7 \times 10^{-7} \ c$	1.07	0.0004	0.1013	0.01294	1.07273

**Fig. 3:**
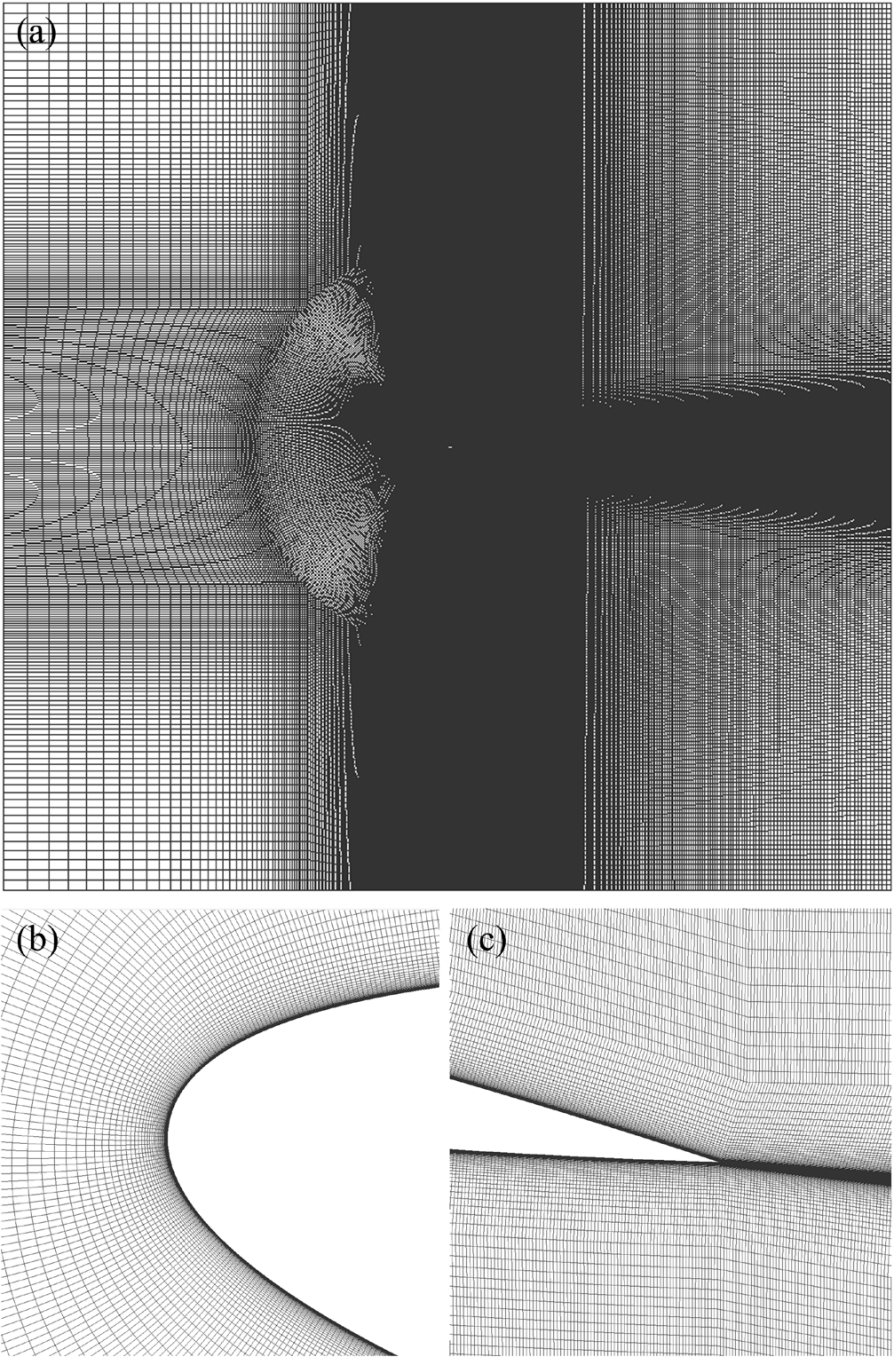
Representation of the intermediate grid at **a** the whole domain, **b** the leading edge, and **c** the trailing edge of a NACA 0012 airfoil. The domain size is $A=30$

Figure 3 shows that the grids were refined in regions with large spatial gradients, that is, around the airfoil and in the wake region. To ensure precise resolution of velocity gradients near the airfoil using the Spalart-Allmaras model, Eça et al. [24] suggested employing a value of $y^{+}=y u_{\tau }/\nu < 1$, where *y*, $\nu$, and $u_{\tau }$ are the height of the first cell, kinematic viscosity, and friction velocity, respectively. Additionally, Eça et al. [24] recommended a value of $y^{+} \simeq 0.1$ for the $k-\omega$ SST model. The values of $\min y^{+}$ and $\max y^{+}$ for different grid resolutions, namely coarsest, coarse, intermediate, and fine, are presented in Table 4. All further results in this study utilized the fine grid, which has a $\max y^{+}$ of approximately 0.1, for all simulations. As presented in Table 4, the growth ratio represents the expansion of adjacent cells perpendicular to the airfoil for the initial 18, 36, 72, and 144 layers from the airfoil for the coarsest, coarse, medium, and fine grids, respectively. Furthermore, Table 4 shows the first grid height, which is twice the distance from the center of the cell next to the airfoil surface to the airfoil. This height was constant for all adjacent cells.

## Results

This section discusses the impact of different boundary conditions and domain sizes on the execution time, velocity and pressure profiles, and lift and drag coefficients.

Section 4.1 first compares the time, $C_{\textrm{d}}$, and $C_{\textrm{l}}$ for four BCs that are usually used for an airfoil simulation. Subsequently, BC-3 is selected among these four conditions to investigate the effect of domain size on $C_{\textrm{d}}$, $C_{\textrm{l}}$, and the simulation time. Additionally, the results of PVBC were compared with those obtained from BC-3 at different domain sizes. We also derive an approximation for $C_{\textrm{d}}$ for $A\rightarrow \infty$, denoted as $C_{\mathrm {d,\infty }}$, along with an approximation for $C_{\textrm{l}}$ for three values of $\alpha$.

In Section 4.2, we study the effect of different boundary conditions on the velocity, pressure, and spurious vorticities inside domains and along boundaries.

Section 4.3 derives a relation between the generated vorticities, lift, and drag using the impulse equation.

Section 4.4 compares the pressure and skin friction coefficients of PVBC and BC-3. We indicate that PVBC allows for a reduction in domain size without losing accuracy, in contrast to BC-3.

### Effect of domain size and boundary conditions on the lift, drag, and execution time

$C_{\textrm{d}}$, $C_{\textrm{l}}$, and execution time for BC-1, BC-2, BC-3, and BC-4 are listed in Table 5 for the baseline case of $\alpha = 10^\circ$ and $A=30$. All simulations were run on the University of Calgary’s high-performance cluster, and no attempt was made to optimize the parallelization. Each simulation was run separately on one compute node with forty cores. Table 5 indicates that $C_{\textrm{l}}$ and $C_{\textrm{d}}$ for BC-2 were significantly different to the other BCs. This table also shows that BC-1 and BC-4 gave very similar results to BC-3. This was true for all *A* and so no further results for these two BCs are shown in the interests of brevity. Since the forces and execution time are very similar for BC-1, BC-3, and BC-4, whereas the longer running BC-2 is less accurate, we will take BC-3 as representative of current practice in applying BCs. Table 6 compares the execution time between BC-3 and PVBC for different values of *A*. Using PVBC increases the execution time compared to BC-3, but we conclude that the reduction in *A* allowed by the PVBC more than offsets the increase in execution time.

**Table 5:** $C_{\textrm{d}}$ and $C_{\textrm{l}}$ for a NACA 0012 airfoil at $\alpha =10^{\circ }$ and $A=30$ for different boundary conditions

Boundary condition	$\varvec{C}_{\textbf{d}}$	$\varvec{C}_{\textbf{l}}$	Time [s]
BC-1	0.01294	1.07273	4,895
BC-2	0.01598	1.05473	5,035
BC-3	0.01294	1.07273	4,928
BC-4	0.01294	1.07273	4,935

**Table 6:** Comparison of the execution time of a NACA 0012 airfoil with BC-3 and PVBC for different domain sizes

A	Number of cells	Time (BC-3) [s]	Time (PVBC) [s]
500	16,320,000	93,399	-
300	14,354,176	47,118	-
100	8,246,272	20,192	-
50	3,825,078	8,198	8,917
30	2,424,832	4,928	5,907
10	1,616,480	3,235	4,464
5	1,193,074	2,257	3,953
3	1,019,570	1,903	3,043

Table 7 compares $C_{\textrm{d}}$, the pressure drag, $C_{\textrm{dp}}$, the frictional drag, $C_{\textrm{df}}$, and $C_{\textrm{l}}$ for BC-3 and PVBC for varying *A* and $\alpha$. Over the large range of *A* in Table 7, $C_{\textrm{df}}$ for BC-3 varies much less than $C_{\textrm{dp}}$; a similar trend was found for BC-1, BC-2, and BC-4. BC-2 results are presented in Table 8 as an example. $C_{\textrm{df}}$ for the PVBC also has a weak dependence on *A*, but it takes a reduction to $A=3$ for the PVBC pressure drag to rise significantly.

**Table 7:** Comparison of force components for a NACA 0012 airfoil at $\alpha =5^{\circ }$, $10^{\circ }$, and $14^{\circ }$ with BC-3 (columns two to five), PVBC (last four columns), and different domain sizes

*A*	*C*_d_	*C*_dp_	*C*_df_	*C*_l_	*C*_d_	*C*_dp_	*C*_df_	*C*_l_
$$\alpha =5^{\circ }$$								
500	0.00902	0.00229	0.00673	0.54905	-	-	-	-
300	0.00903	0.00230	0.00673	0.54895	-	-	-	-
100	0.00907	0.00234	0.00673	0.54849	-	-	-	-
50	0.00913	0.00240	0.00673	0.54778	0.00901	0.00228	0.00673	0.54924
30	0.00922	0.00248	0.00674	0.54686	0.00901	0.00228	0.00673	0.54926
10	0.00963	0.00289	0.00674	0.54267	0.00902	0.00228	0.00674	0.54959
5	0.01025	0.00349	0.00676	0.53743	0.00904	0.00229	0.00675	0.55051
3	0.01109	0.00430	0.00679	0.53182	0.00910	0.00233	0.00677	0.55236
$$\alpha =10^{\circ }$$								
500	0.01219	0.00590	0.00629	1.07648	-	-	-	-
300	0.01223	0.00594	0.00629	1.07633	-	-	-	-
100	0.01239	0.00609	0.00629	1.07550	-	-	-	-
50	0.01262	0.00633	0.00629	1.07424	0.01215	0.00586	0.00629	1.07687
30	0.01294	0.00664	0.00630	1.07273	0.01215	0.00586	0.00629	1.07708
10	0.01449	0.00817	0.00631	1.06529	0.01216	0.00586	0.00629	1.07767
5	0.01680	0.01045	0.00634	1.05672	0.01219	0.00588	0.00631	1.07981
3	0.01990	0.01351	0.00640	1.04820	0.01228	0.00595	0.00633	1.08377
$$\alpha =14^{\circ }$$								
500	0.01802	0.01239	0.00563	1.45162	-	-	-	-
300	0.01807	0.01245	0.00563	1.45142	-	-	-	-
100	0.01835	0.01272	0.00563	1.45048	-	-	-	-
50	0.01877	0.01313	0.00563	1.44910	0.01794	0.01231	0.00563	1.45210
30	0.01932	0.01368	0.00564	1.44733	0.01794	0.01231	0.00563	1.45227
10	0.02206	0.01639	0.00567	1.43952	0.01795	0.01231	0.00563	1.45333
5	0.02617	0.02045	0.00572	1.43164	0.01798	0.01234	0.00564	1.45671
3	0.03178	0.02597	0.00581	1.42590	0.01810	0.01243	0.00567	1.46306

**Table 8:** Comparison of force components for a NACA 0012 airfoil at $\alpha =10^{\circ }$ with BC-2 and different domain sizes

*A*	*C*_d_	*C*_dp_	*C*_df_	*C*_l_
500	0.01241	0.00612	0.00629	1.07524
300	0.01259	0.00629	0.00629	1.07429
100	0.01350	0.00720	0.00630	1.06913
50	0.01476	0.00845	0.00631	1.06181
30	0.01598	0.00966	0.00632	1.05473
10	0.02188	0.01551	0.00637	1.01787
5	0.02908	0.02264	0.00644	0.96901
3	0.03694	0.03041	0.00653	0.90616

Table 9 shows $C_\mathrm {d,\infty }$ and $C_\mathrm {l,\infty }$, the values of $C_{\textrm{d}}$ and $C_{\textrm{l}}$ as $A \rightarrow \infty$. They were calculated using the least square extrapolation for BC-3 and PVBC. The values of $C_{\mathrm {d,\infty }}$ and $C_{\mathrm {l,\infty }}$ are in good agreement for the two BCs implying that either will give accurate results for sufficiently large *A*. $C_{\mathrm {d,\infty }}$ and $C_{\mathrm {l,\infty }}$ that are used in all subsequent equations refer to the values for BC-3, as listed in Table 9.

**Table 9:** $C_\mathrm {d,\infty }$ and $C_\mathrm {l,\infty }$ for a NACA 0012 airfoil at $\alpha =5^{\circ }$, $10^{\circ }$, and $14^{\circ }$ calculated using the least square extrapolation

	$\varvec{C}_{\textbf{d}\varvec{,\infty }}$		$\varvec{C}_{\textbf{l}\varvec{,\infty }}$	
$\varvec{\alpha \,[}^{\varvec{\circ }}\varvec{]}$	BC-3	PVBC	BC-3	PVBC
5	0.00901	0.00900	0.54916	0.54917
10	0.01216	0.01213	1.07670	1.07676
14	0.01793	0.01791	1.45182	1.45186

**Fig. 4:**
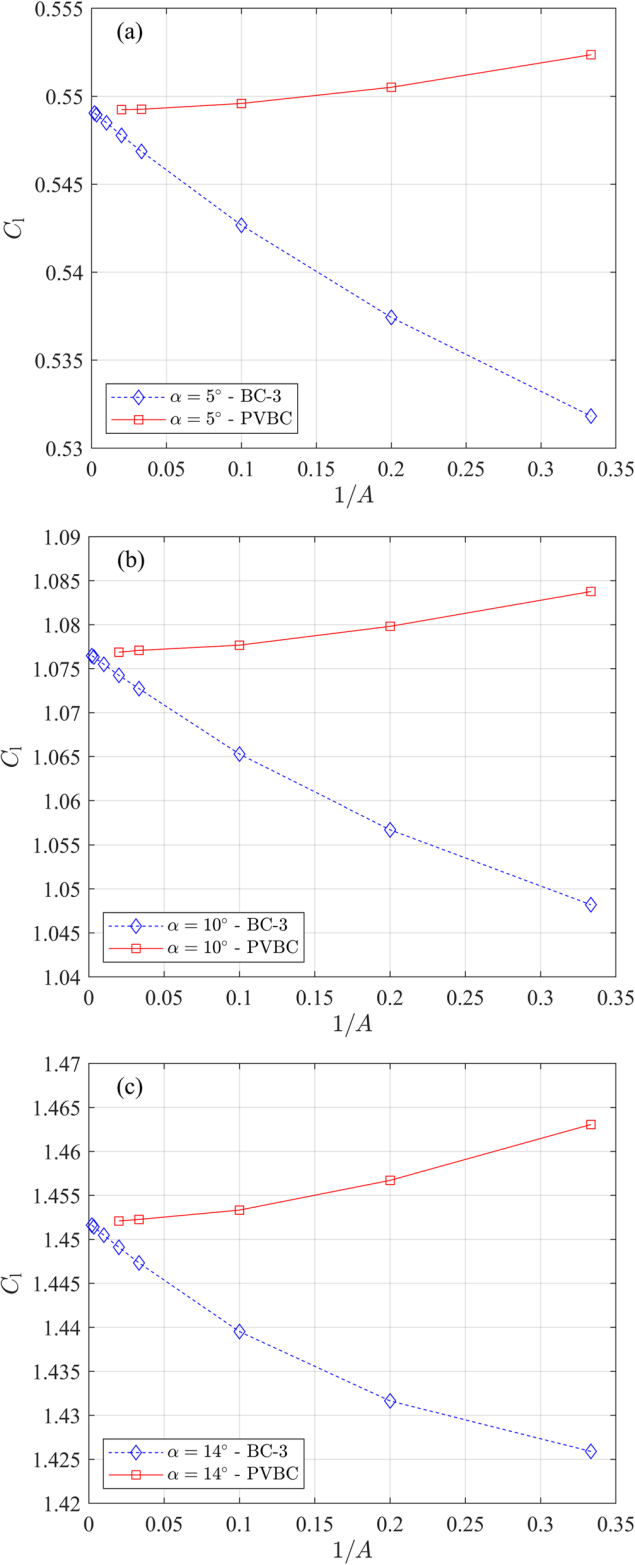
$C_{\textrm{l}}$ for a NACA 0012 airfoil at **a** $\alpha =5^{\circ }$, **b** $\alpha =10^{\circ }$, and **c** $\alpha =14^{\circ }$ with BC-3 and PVBC vs 1/*A*

**Fig. 5:**
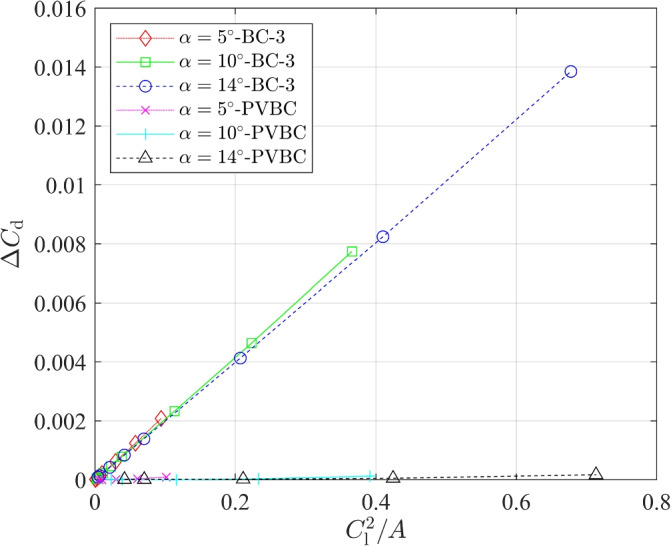
$\Delta C_{\textrm{d}}$ vs ${C^2_{\textrm{l}}}/A$ for a NACA 0012 airfoil at $\alpha =5^{\circ }$, $10^{\circ }$, and $14^{\circ }$ with BC-3 and PVBC

The lift and drag data for BC-3 and PVBC in Table 7 were processed in the following ways. Figure 4 plots the variations of $C_{\textrm{l}}$ with 1/*A*, and Fig. 5 depicts the relationship between $\Delta C_{\textrm{d}}=C_{\textrm{d}}-C_{\mathrm {d,\infty }}$ and $C^2_{\textrm{l}}/A$. First, we note that the relative error in the lift changes less with *A* than does the drag error. Also, it is important that the trend of $C_{\textrm{l}}$ with *A* for BC-3 is opposite that for the PVBC. Since the trend of $C_{\textrm{d}}$ is opposite that for $C_{\textrm{l}}$ for BC-3, the lift-to-drag ratio, which often determines aerodynamic efficiency, has a higher error than either the lift or drag. In the Introduction, it was mentioned that Destarac [13] found a similar behaviour in the pressure drag so the main new result is the near-constancy of $C_{\textrm{df}}$. Destarac [13] also noted that $C_{\textrm{dp}}$ scales with 1/*A* and his least squares fit to the data suggests $C_{\textrm{dp}} \sim C_{\textrm{l}}^2/A$. Using Desterac’s scaling for BC-3 and the three values of $\alpha$ in Table 7, leads to

11 $$\begin{aligned} C_{\mathrm {d,\infty }} \approx C_{\textrm{d}} - 0.0205 C^2_{\textrm{l}}/A, \end{aligned}$$

with an error of less than $1\%$ for $A \ge 5$. Thus, achieving better than a $2 \%$ error in $C_{\textrm{d}}$ at $\alpha = 10^\circ$ using BC-3, requires $A=91$, a value that is rarely used in modern practice. On the other hand, the PVBC requires only $A=5$ for this level of accuracy. It is possible that Eq. (11) is independent of the airfoil shape, which would make it extremely useful.

**Fig. 6:**
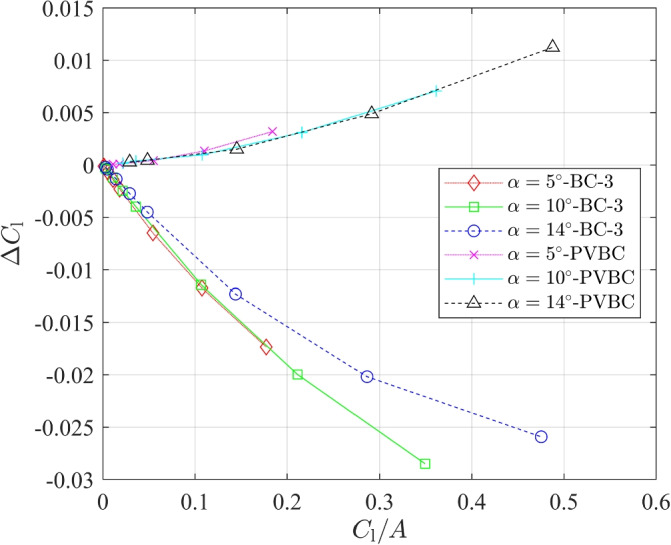
$\Delta C_{\textrm{l}}$ vs $C_1/A$ for a NACA 0012 airfoil at $\alpha =5^{\circ }$, $10^{\circ }$, and $14^{\circ }$ with BC-3 and PVBC

Figure 6 shows the relationship between $\Delta C_{\textrm{l}}=C_{\textrm{l}}-C_{\mathrm {l,\infty }}$ and $C_{\textrm{l}}/A$. Using squares to derive the best fit for PVBC, we obtained

12 $$\begin{aligned} C_{\mathrm {l,\infty }} \approx -0.0341 \frac{C^2_{\textrm{l}}}{A^2}-0.0065 \frac{C_{\textrm{l}}}{A}+C_{\textrm{l}}, \end{aligned}$$

with an error of less than $0.2\%$. Figure 6 shows that it is not possible to derive a similar data fit for BC-3 that uses only $C_{\textrm{l}}$ and *A*. The error in $C_{\textrm{l}}$ is much larger than for PVBC and is of opposite sign.

### Velocity, pressure, and vorticity over the domain

**Fig. 7:**
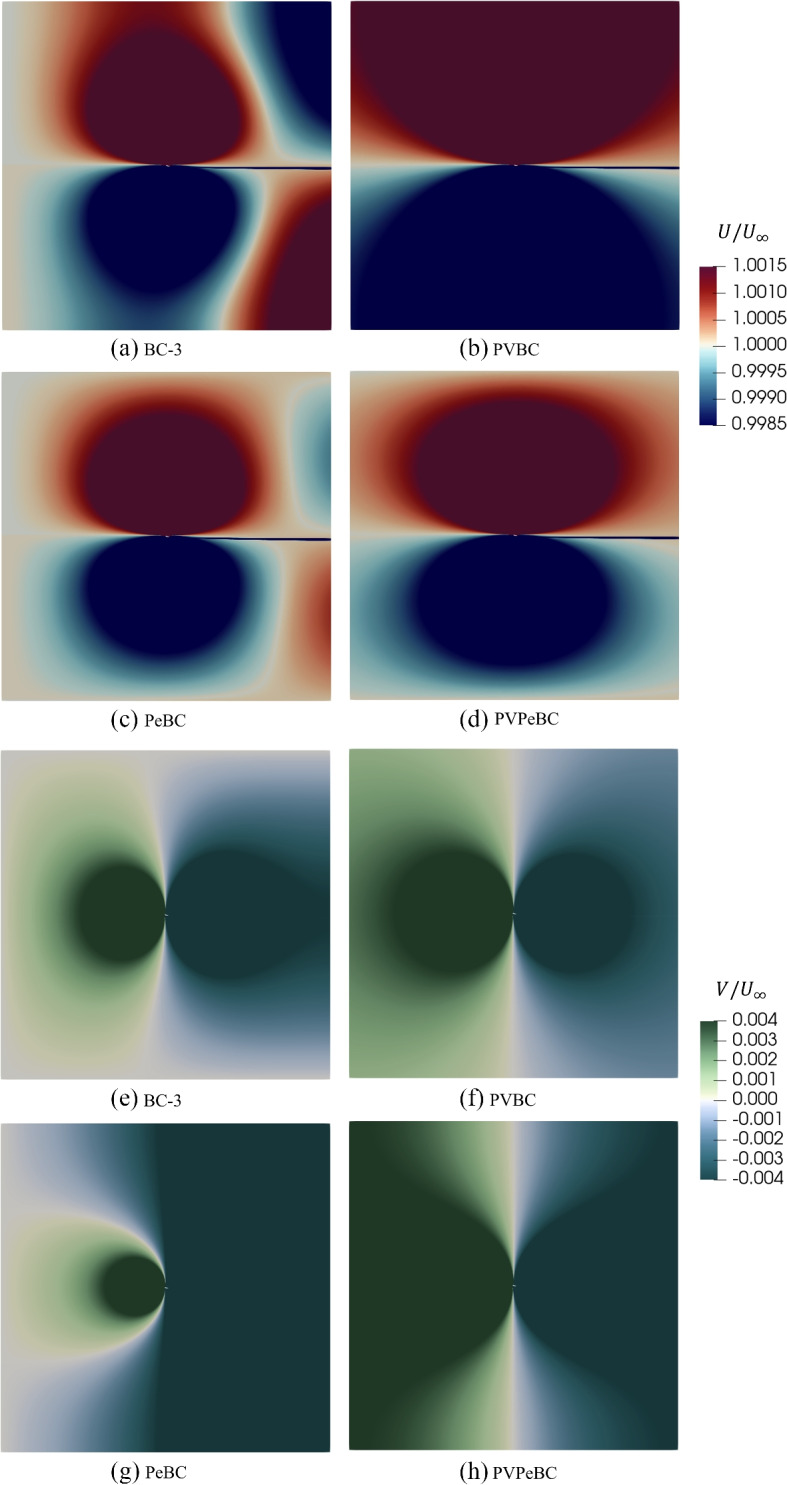
Comparison of the normalized velocity components for different boundary conditions for $A=30$. The parts show the entire domain with the airfoil at the center

**Fig. 8:**
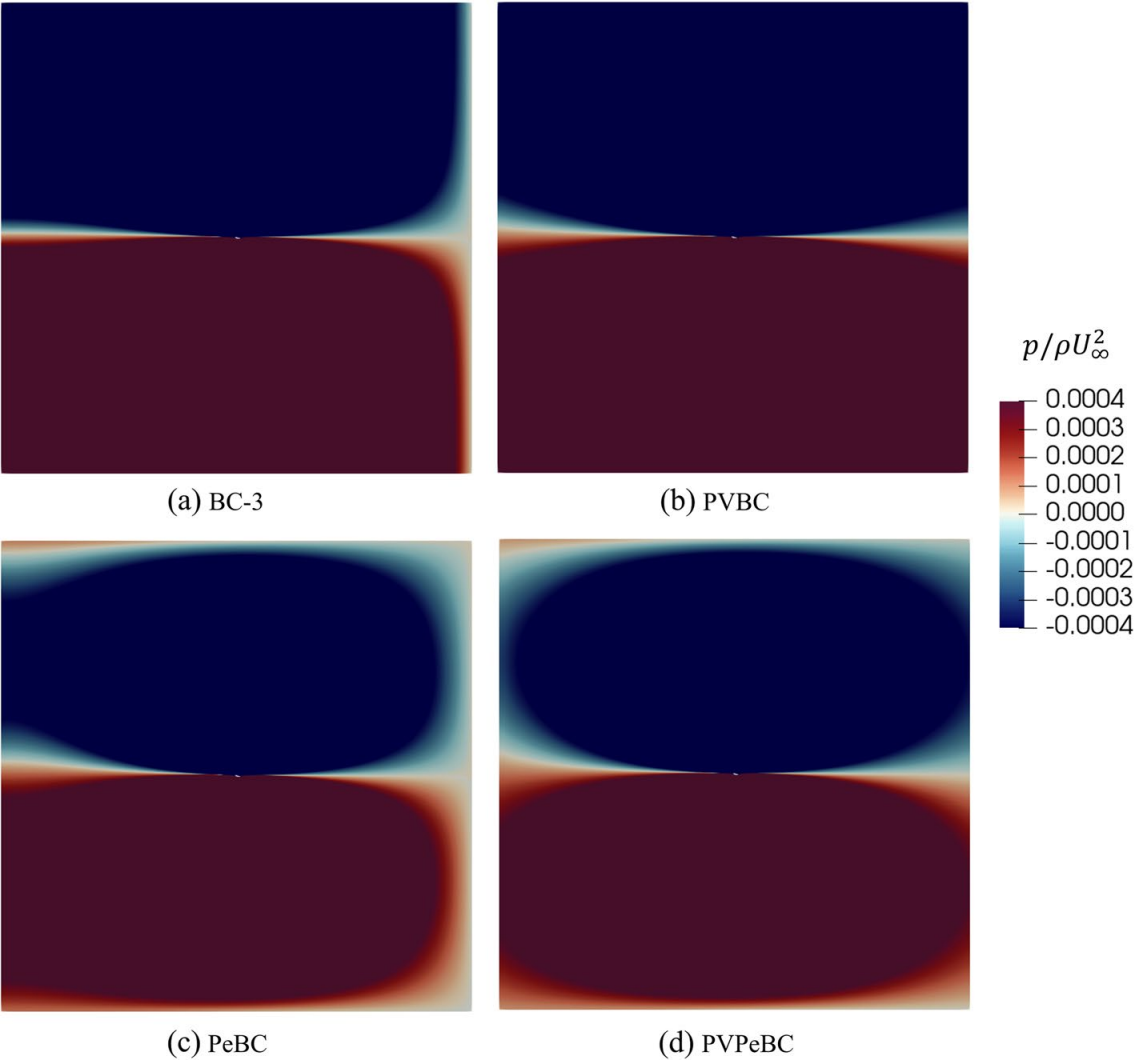
Comparison of the normalized pressure for different boundary conditions for $A=30$

The effects of the different BCs are apparent in the contour plots of the velocities, Fig. 7, and pressure, Fig. 8. We did not consider PeBC and PVPeBC in the previous section on airfoil results, but they are included here to clarify the role of the BCs. Note that all parts of the contour plots show the complete domain. The main differences in the velocities and pressure occur near the boundaries; applying the PVBC Eq. (8) at the outlet makes *u*(*y*) and *v*(*y*) equal to the corresponding inlet *u*(*y*) and $-v(y)$, respectively, even though the normal velocity gradient was set to zero at the outlet. This Neumann condition was used in all BCs, but was just shown to have little effect as part of the PVBC and the same is true for PVPeBC. It does have a big effect, however, for BC-3 where the velocities at the corners, *u*(*A*, *A*) and $u(A,-A)$ have changed sign from those for PVBC. We return to the significance of the corner velocities in discussing the impulse analysis in Section 4.3.

The PVBC velocity at the inlet, Eq. (6), ensures that the inlet pressure matches the outlet pressure. For PeBC, the spurious *U* velocities near the outlet of Fig. 7 part (c) can be attributed to using the zero pressure condition instead of Eq. (10) at the outlet. Furthermore, the zero pressure condition leads to an abrupt pressure drop near the outlet in Fig. 8 parts (a) and (c) in contrast to parts (b) and (d) of the same figure.

**Fig. 9:**
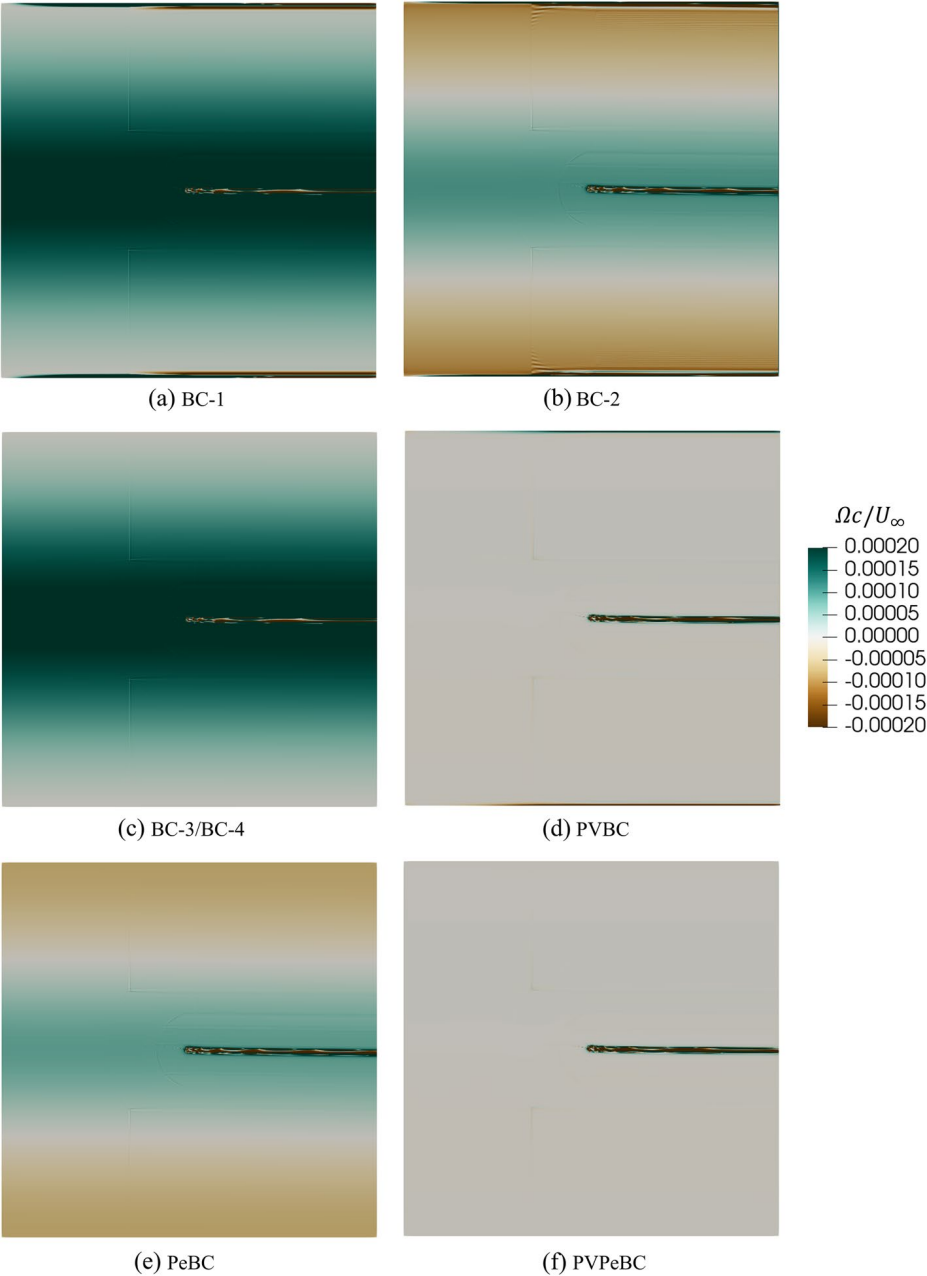
Comparison of normalized vorticity, $\Omega c/U_{\infty }$, for a NACA 0012 airfoil at $\alpha =10^{\circ }$ having $A=30$ and different boundary conditions

Figure 9 for the vorticity outside the boundary layers and wake, is the only one that shows results for all BCs, as they differed in the amount of vorticity generated. This figure also shows the normalized $\Omega$ along the boundaries for different BCs. Only the slip condition, BC-3, and symmetry condition, BC-4, make $\Omega = 0$ at the top and bottom boundaries because the slip/symmetric BC makes $v = 0$ and assigns *U* the same values as that of the first-nearest neighbouring cells of the top and bottom boundaries, so $\partial U/\partial y=0$. BC-1, BC-2, and PVBC produce vorticity along the top and bottom boundaries, but PVBC produces less spurious vorticity compared to BC-1 and BC-2. PVBC, nevertheless, produces positive and negative vorticity along the top and bottom boundaries, respectively, possibly in combination with the Neumann outlet condition as discussed in the next section. Interestingly, the BC that minimizes the spurious vorticity is PVPeBC, showing that the “exact” PeBC is not sufficient on its own to minimize the spurious vorticity but becomes effective when combined with Eq. (9) at the inlet and Eq. (10) at the outlet.

**Fig. 10:**
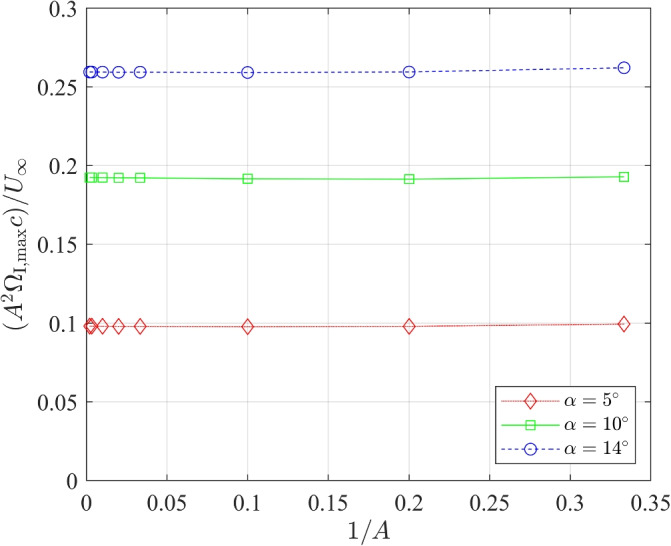
${(A^2 \Omega _\mathrm {{I, max}} c)}/{U_{\infty }}$ vs 1/*A* for a NACA 0012 airfoil at $\alpha =5^{\circ }$, $10^{\circ }$, and $14^{\circ }$ with BC-3

Figure 10 plots the maximum spurious or “boundary-condition” vorticity, $\Omega _{\textrm{BC,max}}$, determined at the inlet; ${(A^2 \Omega _{\textrm{BC, max}} c)}/{U_{\infty }}$ is approximately constant for each $\alpha$ and independent of *A*. This constancy and the scaling of $\Omega _{\textrm{BC,max}}$ on $C_{\textrm{l}}$ means that the effect on the corner velocities mentioned above does not disappear as *A* increases, so Figs. 7 to 9 show results that are qualitatively the same for all *A*.

It is emphasized that the levels of the vorticity contours in Fig. 9 were chosen to be very small to exclude the vorticity in the boundary layers and wake. To put the levels into context, assume that the maximum vorticity, $\Omega _{\textrm{max}}$, in the airfoil boundary layer occurs at the surface. It is then easy to show that $\Omega _{\textrm{max}}c/U_\infty = 2C_{\textrm{f,max}} Re$, where $C_{\textrm{f,max}}$ is the maximum friction coefficient. From the simulation results, $C_{\textrm{f,max}} = 0.0315$ for $\alpha =10^\circ$, from which it follows that $\Omega _{\textrm{max}}c/U_{\infty } = 378,000$. The magnitude of the boundary layer vorticity shed into the wake, $\overline{\Omega }$, is $U_{\infty } / {\delta }_{te}$, where $\delta _{te}$ is the upper surface boundary layer thickness at the trailing edge: $\overline{\Omega }c / U_{\infty } \approx 20$ for $\alpha =10^\circ$. The small values are important, however, because they act over large areas – this is the reason for testing the $A^2$ scaling in Fig. 10 – and influence the momentum balances to be presented below.

### Impulse equations for the lift and drag

Equations (3.55) and (3.56) of Noca [25] give the impulse equation for the force exerted by an incompressible flow on a body contained within an arbitrary CV. We need only the steady state version. He derived the equations by removing the pressure from the conventional Reynolds transport theorem for the momentum equation by using the Navier-Stokes equations. This introduces the vorticity which makes the impulse equations particulary appropriate for the present study as they deal directly with the BC vorticity. If we ignore the viscous and other stresses at the domain boundaries, the equation for the force, $\vec{F}$, becomes

13 $$\begin{aligned} \frac{\vec{F}}{\rho } = \oint _{S} \hat{n} \cdot \big [\frac{1}{2}\vec{U}^2 \vec{I}-\vec{U}\vec{U}-\vec{U} (\vec{x} \times \vec {\Omega })\big ] {\textrm{d}}s. \end{aligned}$$

*S* is the outer boundary of the CV, $\hat{n}$ is the outward-facing unit normal on *S*, $\vec{I}$ is the $m \times m$ unit tensor for an *m*-dimensional flow – $m=2$ here – and ${\textrm{d}}s$ is the magnitude of the vector surface element.

Using the computational domain in Fig. 1 as the CV, for a 2D flow with $\vec{U} = (U_\infty +u, v)$ and $\vec{x}=(x, y)$, the lift and drag derived from Eq. (13) are

14 $$\begin{aligned} \frac{D}{\rho }&=\int _{I} \big [\frac{1}{2} U^2_{\infty }+U_{\infty }u+\frac{1}{2}u^2-\frac{1}{2}v^2 + y(U_{\infty }+u)\Omega \big ]{\textrm{d}}y - \int _{T}(U_{\infty }v + uv +yv\Omega ){\textrm{d}}x\nonumber \\&\quad - \int _{O} \big [\frac{1}{2} U^2_{\infty }+U_{\infty }u+\frac{1}{2}u^2-\frac{1}{2}v^2 + y(U_{\infty }+u)\Omega \big ]{\textrm{d}}y +\int _{B}(U_{\infty }v + uv +yv\Omega ){\textrm{d}}x, \end{aligned}$$

and

15 $$\begin{aligned} \frac{L}{\rho }&= \int _{I} \big [U_{\infty }v +uv - x(U_{\infty }+u)\Omega \big ]{\textrm{d}}y + \int _{T} (\frac{1}{2}U^2_{\infty }+U_{\infty }u+\frac{1}{2}u^2-\frac{1}{2}v^2 + xv\Omega ){\textrm{d}}x\nonumber \\&\quad -\int _{O} \big [U_{\infty }v +uv - x(U_{\infty }+u)\Omega \big ]{\textrm{d}}y - \int _{B} (\frac{1}{2}U^2_{\infty }+U_{\infty }u+\frac{1}{2}u^2-\frac{1}{2}v^2 + xv\Omega ){\textrm{d}}x, \end{aligned}$$

where *I*, *T*, *O*, and *B* indicate the faces of the CV.

In an ideal airfoil simulation, the vorticity is non-zero only in the boundary layers and wake, so we have

16 $$\begin{aligned} \frac{D}{\rho }&= \int _{I} (U_{\infty }u+\frac{1}{2}u^2-\frac{1}{2}v^2){\textrm{d}}y -\int _{T}(U_{\infty }v+uv){\textrm{d}}x\nonumber \\&\quad -\int _{O}\big [U_{\infty }u+\frac{1}{2}u^2-\frac{1}{2}v^2 + y(U_\infty +u)\Omega \big ]{\textrm{d}}y +\int _{B}(U_{\infty }v+uv){\textrm{d}}x, \end{aligned}$$

and

17 $$\begin{aligned} \frac{L}{\rho }&= \int _{I}(U_{\infty }v +uv) {\textrm{d}}y + \int _{T}(U_{\infty }u+\frac{1}{2}u^2-\frac{1}{2}v^2){\textrm{d}}x\nonumber \\&\quad -\int _{O} \big [U_{\infty }v +uv - x(U_{\infty }+u)\Omega \big ]{\textrm{d}}y -\int _{B} (U_{\infty }u+\frac{1}{2}u^2-\frac{1}{2}v^2){\textrm{d}}x. \end{aligned}$$

The first of the integral constraints of Eqs. (1.5) and (1.6) of Liu et al. [26] which are combined as Eq. (9.1.20) of Wu et al. [27]

18 $$\begin{aligned} \int _{O} (U_{\infty }+u) \Omega {\textrm{d}}y=0 \qquad {\text {and}} \qquad \int _{O} \Omega {\textrm{d}}y=0 \end{aligned}$$

removes $\Omega$ from Eq. (17), leaving

19 $$\begin{aligned} \frac{L}{\rho }&= \int _{I}(U_{\infty }v +uv){\textrm{d}}y+\int _{T}(U_{\infty }u+\frac{1}{2}u^2-\frac{1}{2}v^2){\textrm{d}}x\nonumber \\&\quad -\int _{O}(U_{\infty }v +uv){\textrm{d}}y - \int _{B} (U_{\infty }u+\frac{1}{2}u^2-\frac{1}{2}v^2){\textrm{d}}x. \end{aligned}$$

To deal with the quadratic or nonlinear terms involving $u^2$, $v^2$, and *uv*, note that, the PV approximation gives $u(-A,y)=u(A,y)$, $v(-A,y)=-v(A,y)$, $u(x,A)=-u(x,-A)$ and $v(x,A)=v(x,A)$, which reduces some of the quadratic terms. For example, the integral of $u^2$ along T is cancelled by that along B. *uv* is always antisymmetric about $y = 0$ for I and O, and about $x =0$ for T and B, so its integral along each face is zero. Also, from conservation of mass, the sum of the $U_{\infty }u$ and $U_{\infty }v$ terms in Eq. (16) becomes zero. For a sufficiently large *A*, the PV approximation requires the quadratic terms to scale as $C_{\textrm{l}}^2/A$. This scaling suggests a simple origin for the error in drag given by Eq. (11), but we were unable to turn this observation into a prediction for the coefficient (0.0205) in Eq. (11). Neglecting the quadratic terms for sufficiently large *A* gives

20 $$\begin{aligned} \frac{D}{\rho }\approx - \int _{O} y(U_{\infty }+u)\Omega {\textrm{d}}y \xrightarrow [A \rightarrow \infty ]{} -U_{\infty }\int _{O} y\Omega {\textrm{d}}y, \end{aligned}$$

where the asymptotic form is Eq. (1.8) of Liu et al. [26]. See also Eq. (9.1.15) of Wu et al. [27]. Without the quadratic terms, Eq. (19) reduces to Eq. (4) for the lift, where

21 $$\begin{aligned} \Gamma =\int _I v {\textrm{d}}y+\int _T u {\textrm{d}}x -\int _O v {\textrm{d}}y - \int _B u {\textrm{d}}x. \end{aligned}$$

We note, for future purposes, that the PV approximation requires all integrals on the right of Eq. (21) to be equal.

The presence of spurious vorticity for BC-3 alters the lift and drag equations from Eqs. (14) and (15). At I, $u=0$ and $v=0$. At T and B, $v=0$, so Eq. (14) becomes

22 $$\begin{aligned} \frac{D}{\rho } = \int _I y U_{\infty }\Omega {\textrm{d}}y -\int _O\big [U_{\infty } u+\frac{1}{2}u^2-\frac{1}{2}v^2 + y(U_{\infty }+u)\Omega \big ]{\textrm{d}}y, \end{aligned}$$

and, for lift,

23 $$\begin{aligned} \frac{L}{\rho } = U_{\infty }\Gamma + \int _{I}AU_{\infty }\Omega {\textrm{d}}y + \int _{T}\frac{1}{2}u^2{\textrm{d}}x -\int _{O}\big [uv-A(U_{\infty }+u)\Omega \big ] {\textrm{d}}y - \int _{B} \frac{1}{2}u^2 {\textrm{d}}x. \end{aligned}$$

Again, the perturbations, *u* and *v*, must decay asymptotically at least as rapidly as 1/*A* to leave

24 $$\begin{aligned} \frac{L}{\rho } \approx U_{\infty }\Gamma + A\int _{I}U_{\infty }\Omega {\textrm{d}}y+A\int _{O}(U_{\infty }+u)\Omega {\textrm{d}}y. \end{aligned}$$

Now Green’s theorem, which gives the relationship between the area integral of vorticity and circulation, cannot depend on the BCs, so $\Gamma$ in Eq. (24) must equal the difference in circulation of the true bound vortical flow and the circulation due to the spurious vorticity outside the boundary layers and wake. To preserve the KJ equation, the latter circulation then must equal the sum of the two integrals which can only occur if

25 $$\begin{aligned} I_1 =\int _{-A}^A (U_{\infty }+u)\Omega _{BC} {\textrm{d}}y=\int _{I} U_{\infty }\Omega {\textrm{d}}y\ {\text {is invariant with}}\ x. \end{aligned}$$

Invariance also follows from the streamfunction ($\psi$) form of Eq. (25). Define $(U_\infty +u) = \partial \psi / \partial y$ and $v= -\partial \psi /\partial x$, and arbitrarily set $\psi =0$ along B. It follows that

26 $$\begin{aligned} I_1 = \int _{-A}^{A} (U_{\infty }+u)\Omega _{BC} {\textrm{d}}y = \int _0^{2U_\infty A} \Omega _{BC} d \psi, \end{aligned}$$

because BC-3 also makes T a streamline where $\psi (-A, A)=2U_\infty A$ as determined at the inlet. Thus, $I_1$ is invariant. Part of the significance of invariance comes from Eq. (7.4.3) of Batchelor [28] which shows $I_1$ gives the gain in total head of the fluid along T due to the spurious vorticity. From Fig. 10, this gain scales as 1/*A* and so is CV-dependent and is clearly unphysical.

Equation (23) can be rewritten as

27 $$\begin{aligned} L/\rho = U_{\infty }(\Gamma _L-\Gamma _{BC}) + 2 A I_1 = U_{\infty }\Gamma _L, \end{aligned}$$

where $\Gamma _L$ is the lift-generating circulation due to the bound vorticity. Furthermore, the integral in the drag equation, Eq. (20), is altered by the boundary condition vorticity. Define $I_2$ as

28 $$\begin{aligned} I_2=\int _{-A}^A y(U_{\infty }+u)\Omega _{BC} {\textrm{d}}y = \frac{1}{U_\infty }\int _0^{2U_\infty A} \psi \Omega _{BC} d \psi , \end{aligned}$$

where the streamfunction form shows that $I_2$ is also invariant, but only for the flow ahead of the airfoil.

Tables 10 and 11 show the values of the terms in Eqs.(23) and (22), when $\alpha = 10^\circ$ and $A=30$. $D/\rho$ and $L/\rho$ from the impulse equation differ from the OpenFOAM direct determination of airfoil forces by less than $1\%$. Table 11 shows the terms in Eq. (22) evaluated at $x = 5$, 10, 20, and 30. As expected, the difference in the quadratic terms gets smaller with increasing *x* and is absorbed by the *x*-dependence of the integral in Column 5. That integral is, therefore, not invariant downstream of the airfoil and is added to $I_2$ at the inlet to determine $D/\rho$. From Eq. (20), the dominant term in $D/\rho$ gives

29 $$\begin{aligned} -U_{\infty } \int _O y \Omega {\textrm{d}}y = -U_{\infty } \int _{-A}^A y \Omega {\textrm{d}}y = A U_{\infty } [u(A,A)+u(A,-A)] \end{aligned}$$

in combination with the Neumann outlet condition $\partial v/\partial x = 0$. The same condition applied to the vorticity equation gives

30 $$\begin{aligned} \int _{O}\Omega {\textrm{d}}y =\int _{-A}^A \Omega {\textrm{d}}y = -u(A,A)+u(A,-A). \end{aligned}$$

Because the drag and the spurious vorticity are both positive, so are the integrals. Thus, $u(A,-A) \ge 0$, whereas the BS law requires $u(A,-A) \le 0$, so $u(A,-A) = 0$, and $u(A,A) = -u(A,-A)$, which requires $\Omega = 0$. This argument explains the substantial difference in *U* near the corner points in Fig. 7 between part (a) for BC-3 with non-zero $\Omega$ and part (b) for PVBC with $\Omega$ much closer to zero. The behaviour of the corner velocities is related to the cancellation of circulation along T and B for BC-3 which is implied by Fig. 7(a) and is quantified in the next paragraph.

**Table 10:** Comparison of $L/\rho$ between the impulse equation and OpenFOAM results for a NACA 0012 airfoil at $\alpha =10^{\circ }$ having BC-3 and $A=30$. $L/\rho$ from Eq. (19) is 1,421.41866, and $L/\rho$ calculated by OpenFOAM is 1,421.47316

$\varvec{U}_{\varvec{\infty }}\varvec{\Gamma }$	$-\int _{\varvec{I}} \varvec{x}\varvec{(U}_{\varvec{\infty }}\varvec{+u)\Omega } \textbf{d}\varvec{y}$	$\int _{\varvec{T}} \frac{\varvec{1}}{\varvec{2}}\varvec{u}^{\varvec{2}} \textbf{d}\varvec{x}$	$-\int _{\varvec{O}} \varvec{uv}\textbf{d}\varvec{y}$	$\int _{\varvec{O}} \varvec{x(U}_{\varvec{\infty }}\varvec{+u)\Omega } \textbf{d}\varvec{y}$	$-\int _{\varvec{B}} \frac{\varvec{1}}{\varvec{2}}\varvec{u}^{\varvec{2}} \textbf{d}\varvec{x}$
340.86258	540.35038	0.11978	$-0.02323$	540.23760	$-0.12845$

**Table 11:** Comparison of $D/\rho$ between the impulse equation and OpenFOAM results for a NACA 0012 airfoil at $\alpha =10^{\circ }$ having BC-3 and $A=30$. All quantities have units of m^3^s^−1^.  $I_2 = 3.33163$ m^3^s^−1^at the inlet and the direct determination of drag gives $D = 17.14205$ m^3^s^−1^

*x*	$-\int _{\varvec{x}} \varvec{U}_{\varvec{\infty }} \varvec{u} \textbf{d}\varvec{y}$	$-\int _{\varvec{x}} \frac{\varvec{1}}{\varvec{2}}\varvec{u}^{\varvec{2}} \textbf{d}\varvec{y}$	$\int _{\varvec{x}} \frac{\varvec{1}}{\varvec{2}} \varvec{v}^{\varvec{2}} \textbf{d}\varvec{y}$	$-\int _{\varvec{x}} \varvec{y(U}_{\varvec{\infty }}\varvec{+u)\Omega } \textbf{d}y$	$\varvec{D/\rho }$ (Eq. (22))
5	0.00195	$-1.75135$	3.36419	12.16671	17.11313
10	0.00058	$-0.45157$	1.67994	12.54794	17.10852
20	$-0.00020$	$-0.25058$	0.78009	13.24849	17.10944
30	0.01248	$-0.63457$	0.59645	13.83673	17.14272

Columns 2 and 5 in Table 10 suggest the invariance of $I_1$ and this was found to be the case at all *x* to within $1\%$. Again, the quadratic terms are very small, and *L* is dominated by $I_1$ and the $\Gamma$ term, which is approximately one-quarter the magnitude of $L/\rho$. The reason for this is apparent from Table 12 which shows the contributions to $\Gamma$ from the four CV faces for BC-3 and PVBC. As explained above, the contributions are nearly equal for the PVBC, but BC-3 has nearly cancelled the contributions from the inlet, top, and bottom boundaries, leading to the approximately one-quarter contribution to $L/\rho$ from the circulation in the last column of Table 12. The cancellation for T and B is associated with the interaction of BC-3 with the Neumann outflow condition on *v*. It is now clear that this cancellation is directly associated with the generation of $\Omega _{\textrm{BC}}$, but we have not found any direct causal connection that would lead to a prediction of the magnitude of $\Omega _{\textrm{BC}}$.

**Table 12:** Comparison of the circulation between BC-3 and PVBC for a NACA 0012 airfoil at $\alpha =10^{\circ }$ having $A=30$

Boundary condition	$\int _{\varvec{I}} \varvec{v} \textbf{d}\varvec{y}$	$\int _{\varvec{T}} \varvec{u} \textbf{d}\varvec{x}$	$-\int _{\varvec{O}} \varvec{v} \textbf{d}\varvec{y}$	$-\int _{\varvec{B}} \varvec{u} \textbf{d}\varvec{x}$	$\varvec{\Gamma }$
BC-3	0	−0.268	7.508	−0.619	6.621
PVBC	6.931	6.931	6.928	6.931	27.721

One further aspect of the generation of $\Omega _{\textrm{BC}}$ is that the inequality of the contributions to $\Gamma$ shown in Table 12 implies that the irrotational flow has gained strain as well as vorticity. For a rectangular domain, the contributions to $\Gamma$ from the top and bottom boundaries are from $\partial u/\partial y$ and those to the inlet and outlet from $\partial v/\partial x$. Since $\Omega$ must be constant along a streamline in the irrotational flow and $\partial u/\partial y = 0$ at the inlet, and $\partial v/\partial x = 0$ at the outlet, the magnitudes of rotation and strain must be comparable. The dynamics of such a flow may make an interesting study, but one that is obviated if the PVBC is substituted for BC-3.

**Fig. 11:**
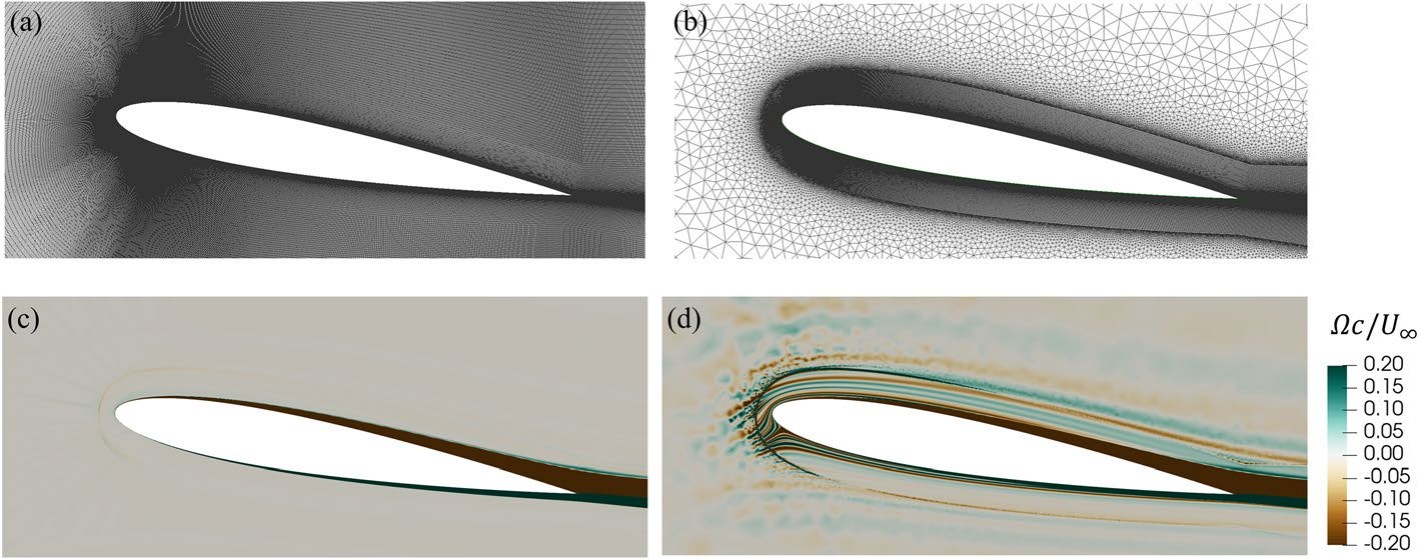
**a** Structured and **b** unstructured grids for a NACA 0012 airfoil at $\alpha =10^{\circ }$ with $A=30$ and BC-3. Note that the grids at the boundary layer are structured for both parts. Normalized vorticity for the **c** structured and **d** unstructured grids

So far we have assumed the spurious vorticity arises only from the BCs. It is possible, however, for it to be generated numerically for any BC, and we found this to be the case in our initial investigations using a structured mesh near the airfoil and an unstructured mesh for the outer flow. The differences in spurious vorticity are shown in Fig. 11 for typical simulations at $\alpha = 10^\circ$ plotted using contour levels much larger than in Fig. 9. Parts (a) and (b) of Fig. 11 show the structured and unstructured grids, respectively. As can be seen in part (c), the structured mesh generates little spurious vorticity, whereas part (d) reveals the presence of randomly generated vorticity in the unstructured mesh. Any vorticity that penetrates the structured grid closer to the airfoil is then convected downstream without change in level, presumably because the numerics of the structured mesh prevent vorticity from being augmented or destroyed. The structured grid reduces the generation of spurious vorticity, enabling a more precise examination of different boundary conditions. For this reason, all other results in this study were obtained using structured meshes.

### Airfoil pressure and skin friction

**Fig. 12:**
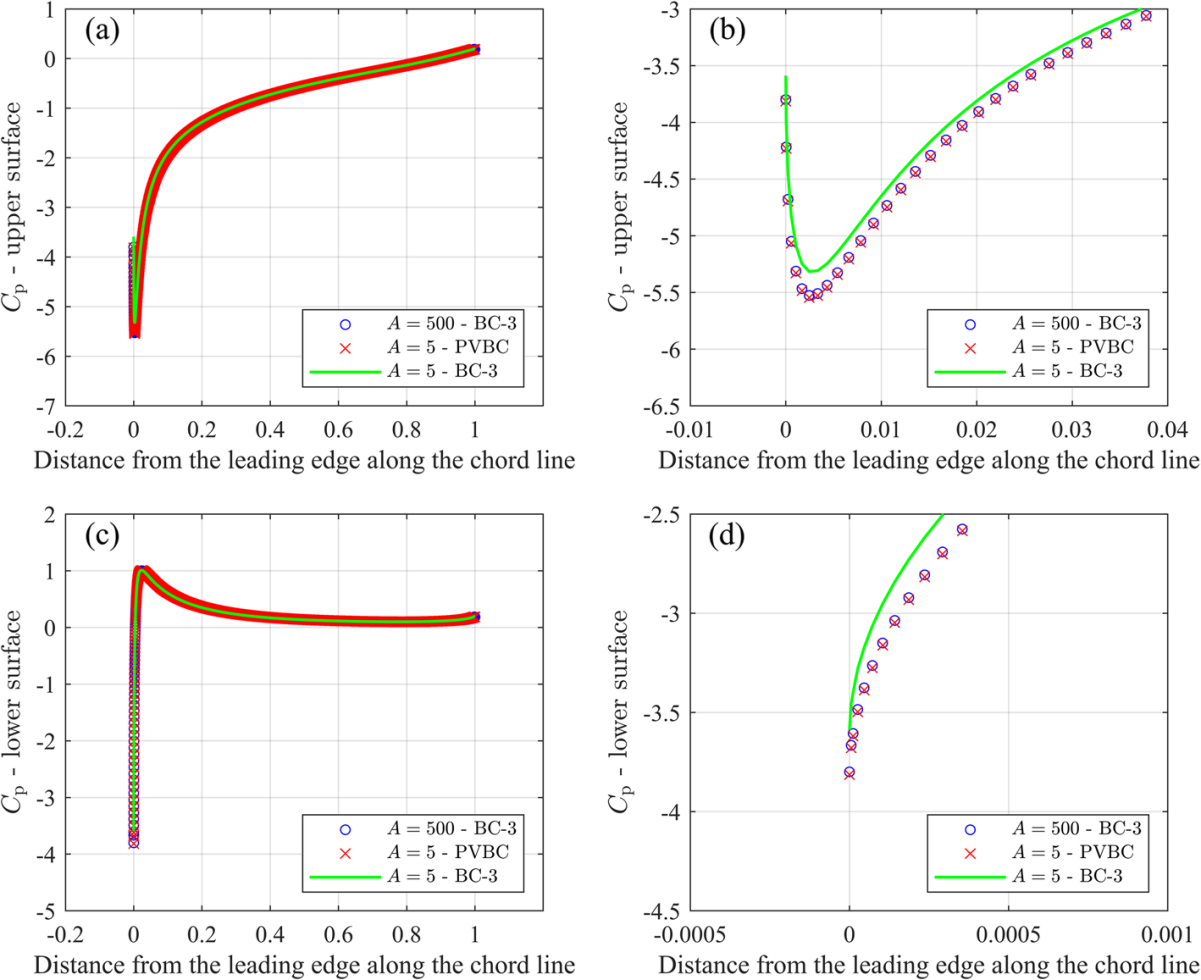
Comparison of $C_{\textrm{p}}$ for airfoil simulations with $A=500$ and BC-3, $A=5$ and PVBC, and $A=5$ and BC-3. **a** $C_{\textrm{p}}$ along the upper surface, **b** Zoom of $C_{\textrm{p}}$ along the upper surface, **c** $C_{\textrm{p}}$ along the lower surface, and **d** Zoom of $C_{\textrm{p}}$ along the lower surface. For parts **b** and **d**, every 5 points are shown

**Fig. 13:**
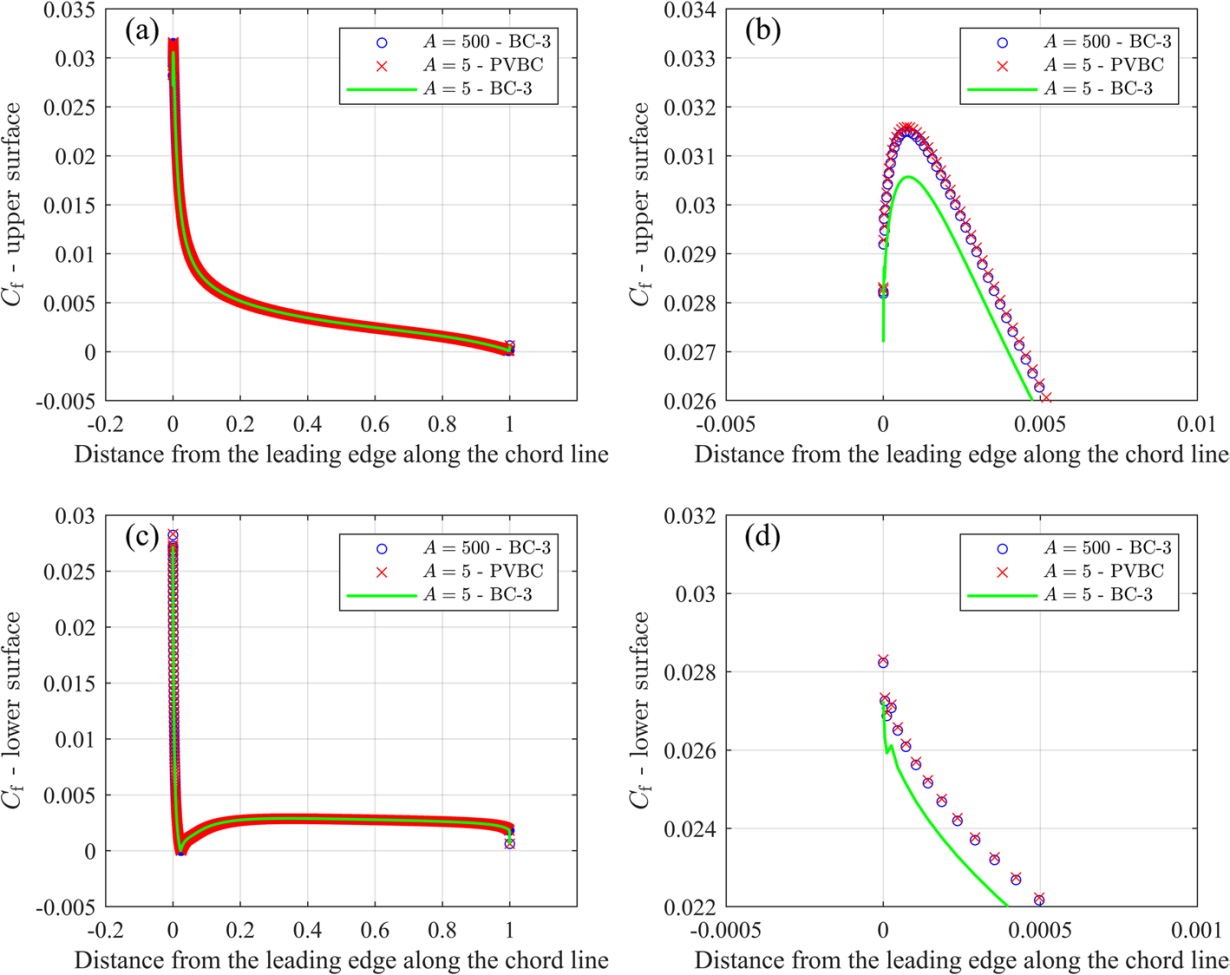
Comparison of $C_{\textrm{f}}$ for airfoil simulations with $A=500$ and BC-3, $A=5$ and PVBC, and $A=5$ and BC-3. **a** $C_{\textrm{f}}$ along the upper surface, **b** Zoom of $C_{\textrm{f}}$ along the upper surface, **c** $C_{\textrm{f}}$ along the lower surface, and **d** Zoom of $C_{\textrm{f}}$ along the lower surface. For parts **b** and **d**, every 5 points are shown

All the results presented so far relate to global quantities like lift and drag, whereas it is important to document the BC effects on the generation of forces around the airfoil. Figures 12 and 13 compare the pressure coefficient, $C_{\textrm{p}}$, and the skin friction coefficient, $C_{\textrm{f}}$, for three cases at $\alpha =10^\circ$: (i) $A=500$ and BC-3, (ii) $A=5$ and PVBC, and (iii) $A=5$ and BC-3. These two figures indicate that using BC-3 and reducing the domain size from *A *= 500 to *A* = 5 results in a significant change for $C_{\textrm{p}}$ and $C_{\textrm{f}}$. In contrast, PVBC with $A=5$ achieves values of $C_{\textrm{p}}$ and $C_{\textrm{f}}$ that are similar to those for the larger domain size of *A* = 500 with BC-3.

## Summary and conclusion

The imposition of boundary conditions (BCs) at the outer edge of the computational domain for a lifting airfoil simulation can never be exact as they depend on the solution. This is in stark contrast to the inner BCs at the airfoil surface where no-slip and impermeability provide exact conditions on the velocity and pressure. This paper considered high Reynolds number ($6\times 10^6$) flow around a NACA 0012 airfoil at three angles of attack: $5^\circ , 10^\circ$, and 14^∘^. We compared four commonly used BCs with the point vortex BC (PVBC). The PVBC provides lift and drag values that are significantly more accurate at any domain size *A* than BC-3, which was found to be typical of the four common BCs that we tested. This improvement in accuracy extends to the local skin friction and pressure on the airfoil surface.

As expected, the choice of domain size and BCs was found to be more critical in relative terms for the drag than the lift. The main outcomes of this study start with the confirmation of the finding by Destarac [13] that the BCs interact with the domain size to generate an (induced) pressure drag that scales with 1/*A* and the square of the lift coefficient. Our second outcome was that the skin friction drag is much less sensitive to the value of *A*. We quantified the domain size required for any desired level of accuracy in the simulation and suggest that with BC-3, *A* must be at least 90 at $\alpha = 10^\circ$ to achieve a 2% accuracy in the drag. This value of *A* is much larger than that used in many studies.

An important manifestation of inexactness was the generation of spurious vorticity within the computational domain. The magnitude of this vorticity was small, but it acts over a large area and influences the momentum balances for a control volume coinciding with the computational domain. All BCs generate spurious vorticity in the supposedly irrotational flow outside the airfoil boundary layers and wake. For the point votex condition, this vorticity was confined to the immediate vicinity of the top and bottom boundaries. The only relevant exact outer BC is the periodic one for a cascade of airfoils, rather than a single airfoil, and it was shown that this BC had to be supplemented with the point vortex boundary condition on the inlet velocity and outlet pressure to produce physically appealing results.

Using the impulse form of the momentum equations, the spurious vorticity throughout the domain was shown to be associated with the cancellation of circulation around the domain boundaries, but we could not formulate an expression for the error in lift that it caused.

The final main outcome of this study was the identification of the interaction of the BCs, particularly the outlet Neumann condition on the velocity with the BCs on the top and bottom boundary. This interaction was associated with the cancellation of circulation along the top and bottom boundaries when BC-3 is used and the generation of spurious vorticity. It appears that one of the main benefits of the PVBC is that it mitigates the effect of the Neumann condition at the outlet velocity, which is almost universally used in airfoil simulations.

As future work, investigating the effect of different turbulence models on boundary conditions and domain sizes would be invaluable. Additionally, it would be interesting to understand whether different airfoil geometries have the same dependence of drag error on the lift and domain size.

## Data Availability

The code used to implement the PVBC and the data produced in this study are available from the corresponding author upon reasonable request.
